# Transcriptome Sequencing and *De Novo* Analysis of *Youngia japonica* Using the Illumina Platform

**DOI:** 10.1371/journal.pone.0090636

**Published:** 2014-03-04

**Authors:** Yulan Peng, Xinfen Gao, Renyuan Li, Guoxing Cao

**Affiliations:** 1 Key Laboratory of Mountain Ecological Restoration and Bioresource Utilization & Ecological Restoration Biodiversity Conservation Key Laboratory of Sichuan Province, Chengdu Institute of Biology, Chinese Academy of Sciences, Chengdu, Sichuan, People's Republic of China; 2 Forest College, Sichuan Agricultural University, Ya'an, Sichuan, People's Republic of China; The Centre for Research and Technology, Hellas, Greece

## Abstract

*Youngia japonica*, a weed species distributed worldwide, has been widely used in traditional Chinese medicine. It is an ideal plant for studying the evolution of Asteraceae plants because of its short life history and abundant source. However, little is known about its evolution and genetic diversity. In this study, *de novo* transcriptome sequencing was conducted for the first time for the comprehensive analysis of the genetic diversity of *Y. japonica*. The *Y. japonica* transcriptome was sequenced using Illumina paired-end sequencing technology. We produced 21,847,909 high-quality reads for *Y. japonica* and assembled them into contigs. A total of 51,850 unigenes were identified, among which 46,087 were annotated in the NCBI non-redundant protein database and 41,752 were annotated in the Swiss-Prot database. We mapped 9,125 unigenes onto 163 pathways using the Kyoto Encyclopedia of Genes and Genomes Pathway database. In addition, 3,648 simple sequence repeats (SSRs) were detected. Our data provide the most comprehensive transcriptome resource currently available for *Y. japonica*. C_4_ photosynthesis unigenes were found in the biological process of *Y. japonica*. There were 5596 unigenes related to defense response and 1344 ungienes related to signal transduction mechanisms (10.95%). These data provide insights into the genetic diversity of *Y. japonica*. Numerous SSRs contributed to the development of novel markers. These data may serve as a new valuable resource for genomic studies on *Youngia* and, more generally, Cichoraceae.

## Introduction

Cichorioideae, one of the most evolved eudicot subfamilies, comprises approximately 2300 species [1] that can adapt to different environments. Some species exhibit weed evolution [2]. Many species of Cichorioideae are used as traditional vegetables, herbs, and fodder for animals. *Youngia japonica* L. (oriental false hawksbeard) is a weed species originated in Asia, but is now world-widely distributed. *Youngia japonica* is used as a Chinese traditional medicine because of its guaiane-type sesquiterpene and other constituents that exhibit strong antiallergic, antioxidant, and antitumor activities [3]–[5]. However, studies about the DNA or protein sequences of this species are limited.

Asteraceae is one of the four largest families in angiosperms and one of the most evolutionary groups. However, transcriptome analyses or expressed sequence tags (ESTs) of Asteraceae species, including only very few species *Chrysanthemum nankingense*, *Cynara cardduncyulus*, *Centaurea solstitialis*, and *Cirsium arvense* (http://www.ncbi.nlm.nih.gov/dbEST/dbEST_summary.html), are insufficient [6]–[9]. Although RNA seq-based transcriptome analysis of *Lactuca sativa* (a member in tribe Cichoraceae) leaves infected by the fungal necrotroph *Botrytis cinerea* has been conducted [10], a comprehensive description of its transcriptome remains unavailable. Thus, biological and evolution studies of morphologically unique and economically important plants of Cichoraceae are limited. *Youngia japonica* is an ideal plant for studying the evolution of Asteraceae plants because of its short life history, abundant source, and rich genetic diversity [1], [11], [12]. Sequencing of large genomes is highly expensive, thus transcriptome analysis is a useful and cost-effective method of discovering new genes, and provides information on gene expression, gene regulation, and amino acid content of proteins [13]–[15].

In this study, we examined the genetic diversity of *Y. japonica* using *de novo* transcriptome sequencing, and investigated the mechanism by which this species adapts to different environments.

## Materials and Methods

### Plant Materials and RNA Extraction

All materials were obtained from Chengdu Institute of Biology, Chinese Academy of Sciences, and naturally grown in flowerpots (Zhongbei, Chengdu, Sichuan, China). The voucher of *Youngia japonica* is Peng2013. All vouchers of samples in our experiments are deposited in the Herbarium of the Chengdu Institute of Biology (CDBI). This species was identified according to Flora of China [1]. The whole plants (above ground parts, including leaves, stems and flowers) were immediately frozen and stored in liquid nitrogen until analysis. Total RNA was extracted from these materials using an EASY spin microRNA Rapid extraction kit (Aidlab Biotechnologies Co., Ltd., China). The quality and quantity of total RNA were analyzed using an Ultrasec TM 2100 pro UV/Visible Spectrophotometer (Amersham Biosciences, Uppsala, Sweden), gel electrophoresis, and Agilent G2939A (Agilent RNA 6000 Nano Kit). Equal quantities of high-quality RNA from each material were pooled for cDNA synthesis.

### mRNA-seq Library Construction for Illumina Sequencing

The mRNA-seq library was constructed using an mRNA-Seq Sample Preparation Kit (Cat# RS-930-1001, Illumina Inc., San Diego, CA, USA) following the manufacturer's instructions. The poly-(A) mRNA was isolated from the total RNA samples with poly-T oligo-attached magnetic beads. The mRNA was fragmented by an RNA fragmentation kit (Ambion, Austin, TX, USA) before cDNA synthesis to avoid priming bias. The cleaved RNA fragments were transcribed into first-strand cDNA using reverse transcriptase (Invitrogen, Carlsbad, CA, USA) (Invitrogen) and random hexamerprimers, followed by second-strand cDNA synthesis using DNA polymerase I and RNase H (Invitrogen). The double-stranded cDNA was end-repaired using T4 DNA polymerase (NEB), Klenowfragment (NEB), and T4 polynucleotide kinase (NEB). A base addition using Klenow 39 to 59 exo-polymerase (NEB) to prepare the DNA fragments for ligation to the adaptors. The products of ligation reaction were purified using a MinElute PCR Purification Kit (QIAGEN, Dusseldorf, Germany) (QIAGEN) following the manufacturer's instructions, and eluted in 10 µL of QIAGEN EB buffer. The eluted adaptor-ligated fragments of the ligation reaction were separated by size on an agarose gel to select a size range of templates for downstream enrichment. The desired range of cDNA fragments was excised and retrieved using a Gel Extraction Kit (Axygen Biosciences, Central Avenue Union City, CA, USA). PCR was performed for the selective enrichment and amplification of the cDNA fragments using Phusion Master Mix (NEB) with two primers (i.e., PCR Primer PE 1.0 and PCR Primer PE 2.0) supplied by an mRNA-Seq Sample Preparation Kit (Illumina). The amplified products were purified using a QIAquick PCR Purification Kit (QIAGEN) following the manufacturer's instructions, and eluted in 30 µL of QIAGEN EB buffer. Libraries were prepared from a 150 bp to 200 bp size selected fraction following adaptor ligation and agarose gel separation. After accurate quantitation (Qubit) of the samples the front steps processed, bridge PCR was performed on the surface of the flow cell to amplify DNA fragments as a single DNA molecule cluster (this process was carried out in the Cluster Station).The flow cell was then transferred into Hi-Seq for sequencing (HiSeqTM 2000 sequencing platform). Data analysis and base calling were performed using Illumina instrument software.

### Sequence Data Analysis and Assembly

The raw reads were evaluated with CycleQ20. The qualified reads were extended into contigs with Trinity software through the overlap between the sequences. Then the contigs were connected into transcript sequences, according to paired-end information of the sequences, which recovers full-length transcripts across a broad range of expression levels, with sensitivity similar to methods that rely on genome alignments [16]. The overlap settings used for this assembly were 24 bp and 80% similarity, group pairs distance was set to 500 (maximum length expected between fragment pair), with all the other parameters set to their default values. We selected the longest transcriptions from the potential assembled component alternative splicing transcripts as unigene sequences of our samples. We quantified transcript levels in reads per kilobase of exon mode per million mapped reads (RPKM) [17]. The RPKM measure of read density reflects the molar concentration of a transcript in the starting sample by normalizing for RNA length and for the total read number in the measurement.

### Sequence Annotation

The optimal assembly results were chosen according to the assembly evaluation. Then, clustering analysis was performed to achieve a unigene database, which comprised the potential alternative splicing transcripts.

The assembled sequences were compared against the National Center for Biotechnology Information (NCBI) non-redundant protein (Nr) database, NCBI non-redundant nucleotide sequence (Nt) database, and Swiss-Prot database using BlASTn (version 2.2.14) with an E-value based on an E-value of less than 10^−5^. Gene names were assigned to each assembled sequence based on the best BLAST hit (highest score). Searches were limited to the first 10 significant hits for each query to increase computational speed. Open reading frames (ORFs) were predicted using the “getorf” program of EMBOSS software package [18], with the longest ORF extracted for each unigene.

To annotate the assembled sequences with gene ontology (GO) terms describing biological processes, molecular functions, and cellular components, the Swiss-Prot BLAST results were imported into Blast2GO [19], [20], which is a software package that retrieves GO terms and allows gene functions to be determined and compared. These GO terms were assigned to query sequences, and produced a broad overview of groups of genes catalogued in the transcriptome for each of the three ontology vocabularies, namely, biological processes, molecular functions, and cellular components. The obtained annotation was enriched and refined using ANNEX [21], [22].

The unigene sequences were also aligned to the Clusters of Orthologous Group (COG) database to predict and classify functions [23]. Kyoto Encyclopedia of Genes and Genomes (KEGG) pathways were assigned to the assembled sequences using the online KEGG Automatic Annotation Server (http://www.genome.jp/kegg/kaas/). The bi-directional best hit method was used to obtain KEGG Orthology (KO) assignment [24]. The output of KEGG analysis included KO assignments and KEGG pathways that were populated with the KO assignments.

EST-simple sequence repeat (SSR) Detection

SSR Identification Tool (http://www.gramene.org/db/markers/ssrtool) was used to identify SSRs in a given sequence [25], [26]. The parameters were adjusted to identify perfect di-, tri-, tetra-, penta-, and hexa-nucleotide motifs with a minimum of six, five, four, four, and four repeats, respectively. The report of this search included the total number of sequences containing SSRs among the submitted unigenes, sequence ID, SSR motifs, number of repeats (di-, tri-, tetra-, penta-, and hexanucleotide repeat units), repeat length, SSR starts, and SSR ends [27]–[29].

## Results

### *Youngia japonica* Transcriptome Sequencing and *De Novo* Assembly

**Figure 1:**
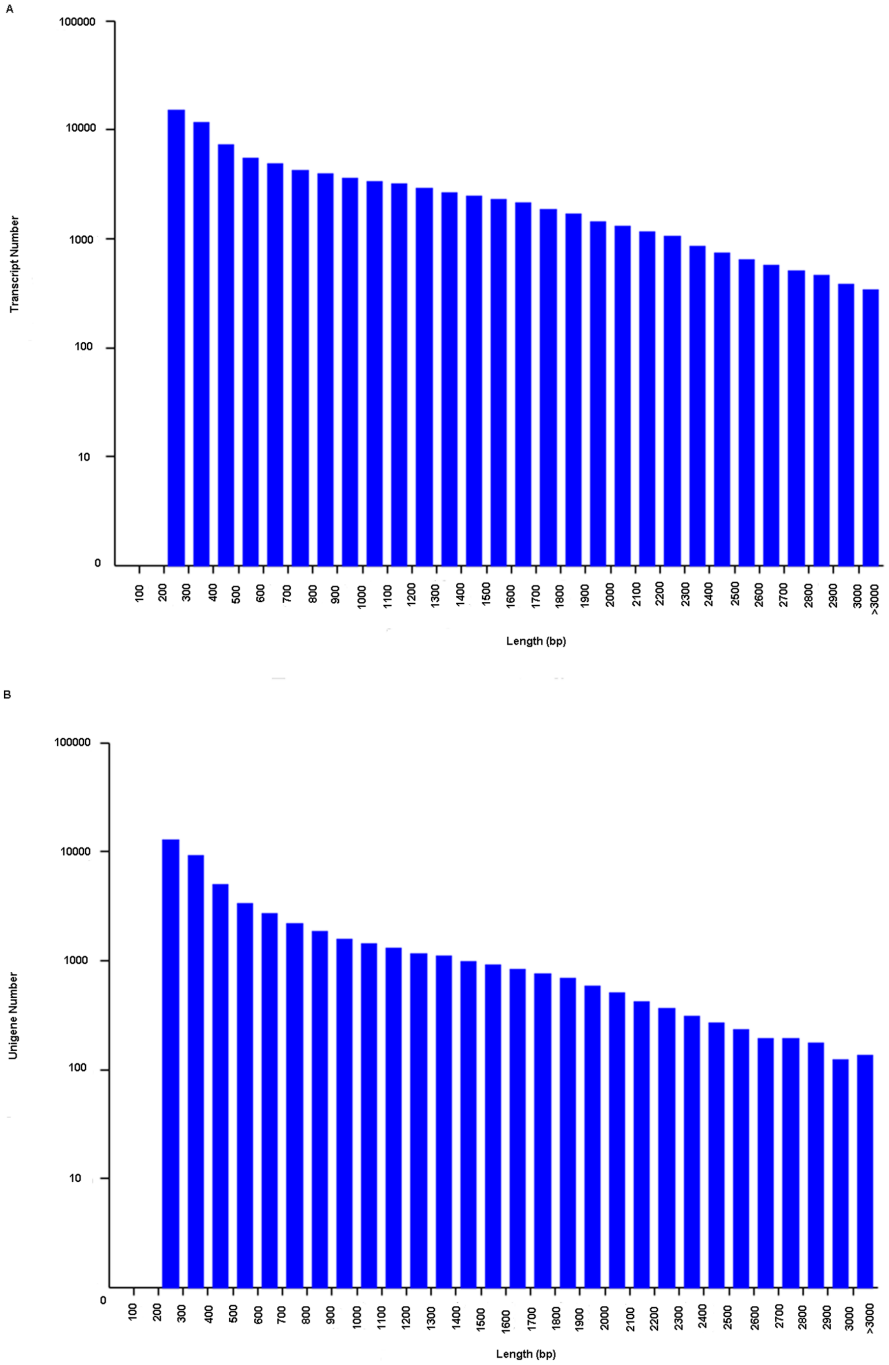
Overview of the *Youngia japonica* transcriptome assembly. (A) Size distribution of contigs; (B) size distribution of unigenes.

Sequence analysis and assembly were performed to obtain a global overview of the transcriptome and gene activities of *Y. japonica* at the nucleotide level. A mixed cDNA sample representing flowers and different tissues of *Y. japonica* was prepared and sequenced using an Illumina Genome Analyzer. The sequenced sample yielded 21,847,909 independent reads (4,412,858,107 bp) from either end of a cDNA fragment with 100% Q20 bases. An overview of the sequencing is presented in Table 1. The high-quality reads produced in this study were deposited in the NCBI SRA database (accession number: SRR1002945). Using the Trinity *de novo* assembly program, next-generation short-read sequences were assembled into 89564 contigs, with an N50 length of 1,497 bp and a mean length of 992.6 bp. The distribution of contigs is shown in Fig. 1A. A total of 33,975 contigs coded for transcripts longer than 1 kb, and 10220 contigs coding for transcripts longer than and 2 kb, respectively. The contigs were subjected to cluster and assembly analyses. A total of 51,850 unigenes were obtained, among which 13,518 genes (26.07%) were greater than 1 kb. The length distributions of the unigenes are shown in Fig.1B. The results show that more than 25,082 unigenes (48.36%) were greater than 500 bp.

**Table 1:** Summary of sequencing and *de novo* assembling of plant transcriptome in *Youngia japonica*.

Transcript Length	Total Number	Percentage
200–300	14937	16.68%
300–500	18703	20.88%
500–1000	21949	24.51%
1000–2000	23755	26.52%
2000+	10220	11.41%
Total number	89564	
Total length	88908044	
N50 length	1497	
Mean length	992.6761199	

### Functional Annotation

We used several complementary approaches to annotate the assembled sequences. The unigenes were annotated by aligning with the deposited ones in diverse protein databases, including Nr database, Nt database, UniProt/Swiss-Prot, KEGG, COG of proteins, and UniProt/TrEMBL. The best one was selected from the matches with an E-value of less than 10^−5^. The overall functional annotation is depicted in Table 2. First, a sequence similarity search was conducted against the NCBI Nr and Nt databases and Swiss-Prot protein database using the Basic Local Alignment Search Tool (BLAST) algorithm specifying E-values less than 10^−5^. There were 36,517 unigenes matched in the Nr database, and 27,929 significantly matched in the Nt database and 28,594 were similar to proteins in the Swiss-Prot database. A total of 36,310 unigene matches were found in the TrEMBL database. A total of 37,734 unigenes were successfully annotated in Nr, Nt, Swiss-Prot, KEGG, GO, COG, and TrEMBL (Table 2).

**Table 2:** Functional annotation of the *Youngia japonica* transcriptome.

Anno Database	Annotated Number	300≤length<1000	length≥1000
OG Annotation	12269	4843	5639
GO Annotation	30610	13968	11594
KEGG Annotation	9125	4084	3179
Swissprot Annotation	28594	12826	11253
TrEMBL Annotation	36310	17277	12902
Nr Annotation	36517	17388	12889
Nt Annotation	27929	12052	11356
All Annotated	37734	17991	12968

### GO Classification

**Figure 2:**
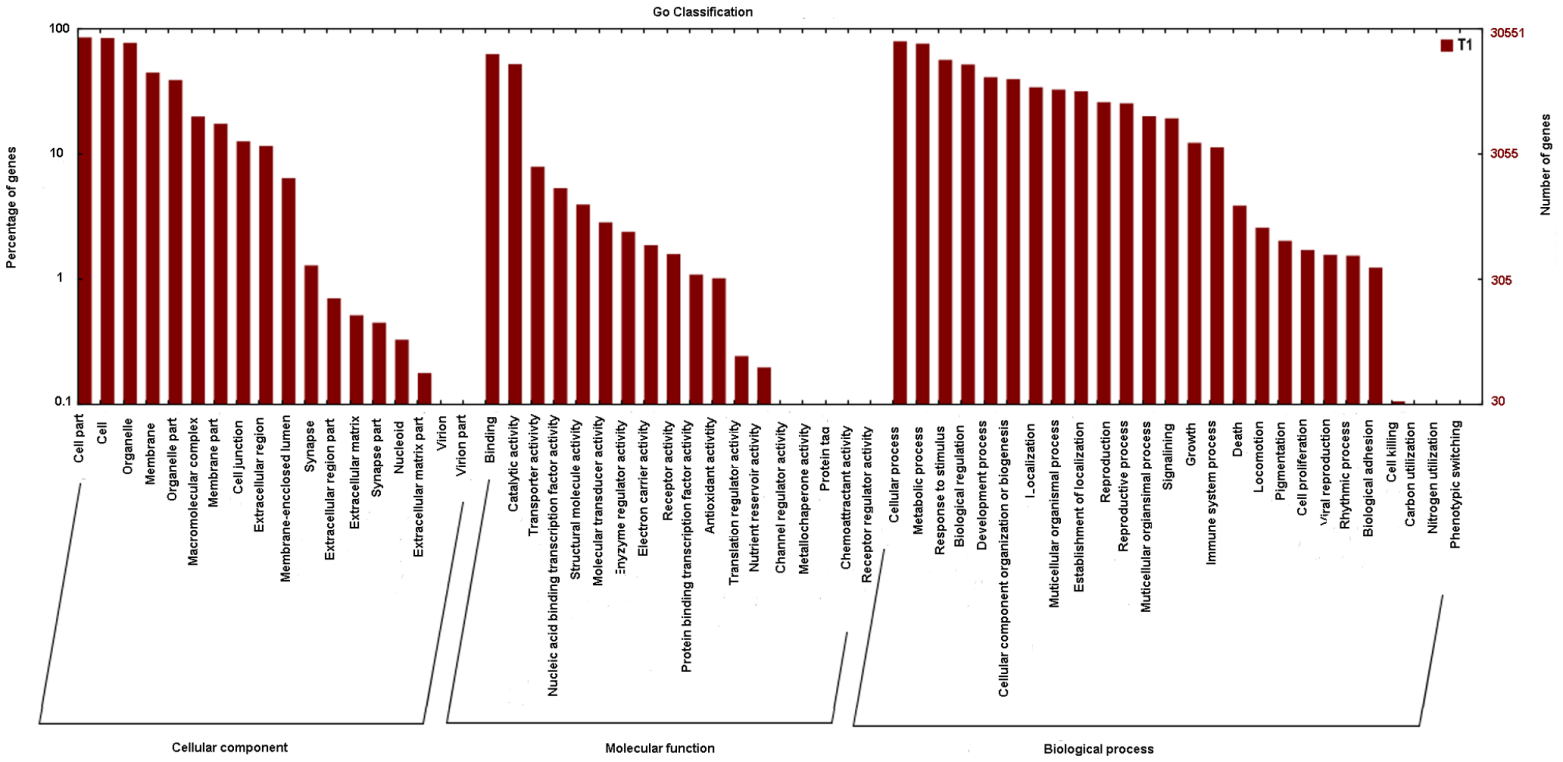
Functional annotation of assembled sequences based on gene ontology (GO) categorization.

GO classification based on sequence homology revealed that 30,610 out of the assembled unigenes were categorized into 62 functional groups in *Y. japonica* (Fig. 2). The three major categories (biological process, cellular component, and molecular function) were assigned to 176,128, 123,982, and 44,253 GO terms, respectively. In the “biological process” category, the unigenes related to “metabolic processes” (76.65%) and “cellular processes” (80.03%), “response to stimulus” (56.86%), “biological regulation” (52.22%), “developmental process” (41.29%), “cellular component organization or biogenesis” (39.97%), “localization” (5.96%), and “multicellular organismal process” (32.88%) were predominant. The “cell parts” (86%) and “cell” (85.10%), “organelle” (77.88%), “membrane” (45.04%), and “organelle part” (39.23%) were found to be the most abundant classes. Under the “molecular function” category, the majority of unigenes were involved in “binding” (63.26%) and “catalytic activities” (52.65%) (Fig. 2).

### COG Classification

**Figure 3:**
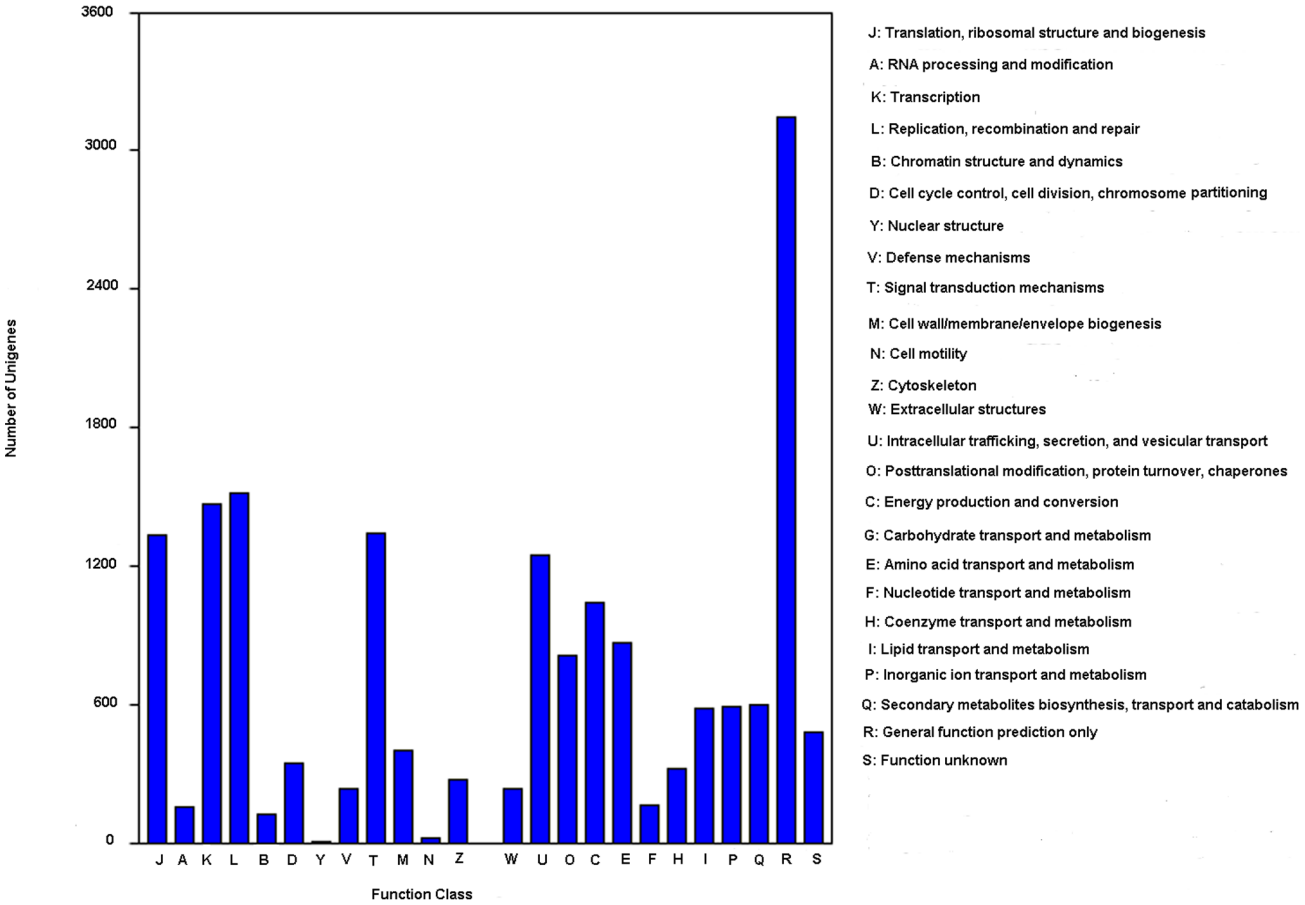
Clusters of orthologous group (COG) classification.

COG classification of 17,296 Nr hits indicated that 12,269 unigenes were clustered into 25 functional categories (Fig. 3). “general function prediction only” (25.64%) was found to be the major COG category, followed by “replication, recombination, and repair” (12.37%), “transcription” (11.99%), “signal transduction mechanisms” (10.95%), “translation, ribosomal structure, and biogenesis”(10.87%), “posttranslational modification, protein turnover, chaperones” 10.16%), and “carbohydrate transport and metabolism”(8.47%) (Fig. 3).

### KEGG Classification

**Figure 4:**
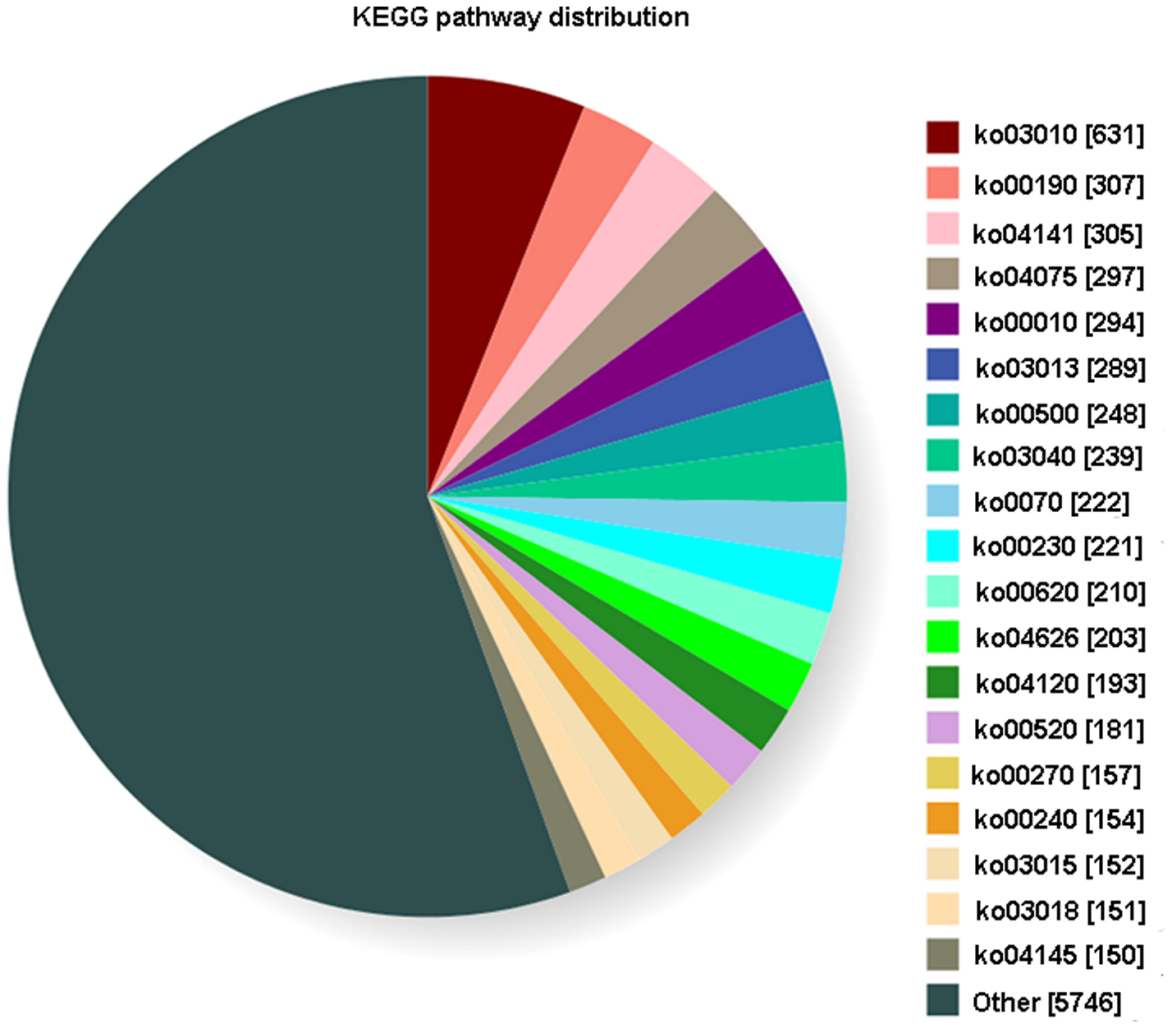
KEGG biochemical mappings for *Youngia japonica*.

Analysis of unigenes through KEGG showed that 9,125 unigenes were assigned to 162 pathways. The major pathways containing hundreds of unigenes were ribosome (ko03010) (631 unigenes, accounting for 6.09%), mRNA surveillance pathway (ko03015) (152, 1.47%), RNA degradation (ko03018) (151, 1.46%), RNA transport (ko03013) (289, 2.79%), protein processing in endoplasmic reticulum (ko04141) (305, 2.94%), plant hormone signal transduction (ko04075) (297, 2.87%), glycolysis/gluconeogenesis (ko00010) (294, 2.84%), starch and sucrose metabolism (ko00500) (248, 2.39%), carbon fixation in photosynthetic organisms (ko00710) (222, 2.14%), purine metabolism (ko00230) (221, 2.14%), spliceosome (ko03040) (239, 2.30%), plant–pathogen interaction (ko04626) (203,1.9%), ubiquitin-mediated proteolysis (ko04120) (193, 1.86%), amino sugar and nucleotide sugar metabolism (ko00520) (181, 1.75%), cysteine and methionine metabolism (ko00270) (157, 1.52%), primidine metabolism (154, 1.49%) (ko00240), and phagosome (ko04145) (150, 1.45%) (Fig. 4).

### SSR Discovery

The 10,251 unigene sequences were submitted to an online service to explore SSR profiles in the unigenes of *Y. japonica*. MISA analysis (MIcroSAtellite, http://pgrc.ipk-gatersleben.de/misa/) identified 3,648 putative microsatellites from 3,092 unigenes (30.16%). Six types of SSRs were found, among which 556 sequences contained more than one SSR. Tri-nucleotide repeat motif was the most abundant, accounting for 40.193%, followed by mono-nucleotide (36.76%), di-nucleotide repeat motif (16.15%), compound SSR (5.56%), tetranucleotide (1.23%), and penta-nucleotide (1.12%) (Table 3).

**Table 3:** Summary of simple sequence repeat (SSR) types in *Youngia japonica*.

Searching item	Numbers
Total number of sequences examined	10251
Total number of identified SSRs	3648
Number of sequences containing more than one SSR	564
Number of SSRs present in compound formation	203
Mono-nucleotide	1341
Di-nucleotide	589
Tri-nucleotide	1466
Tetra-nucleotide	45
Penta-nucleotide	4
Hexa-nucleotide	0

## Discussion

The mean unigene length of *Y. japonica* (795 bp) was longer than that of *Chrysanthemum nankingense* (585 bp). The N50 length of *Y. japonica* transcriptome was significantly longer (1497 bp) (Table 1) than that of *Cynara cardduncyulus* transcriptome (951 bp), which suggests that the quality of our assembly was high [8], [9]. N50 length is commonly used for assembly evaluation, and a high number suggests high quality assembly [30].

**Figure 5:**
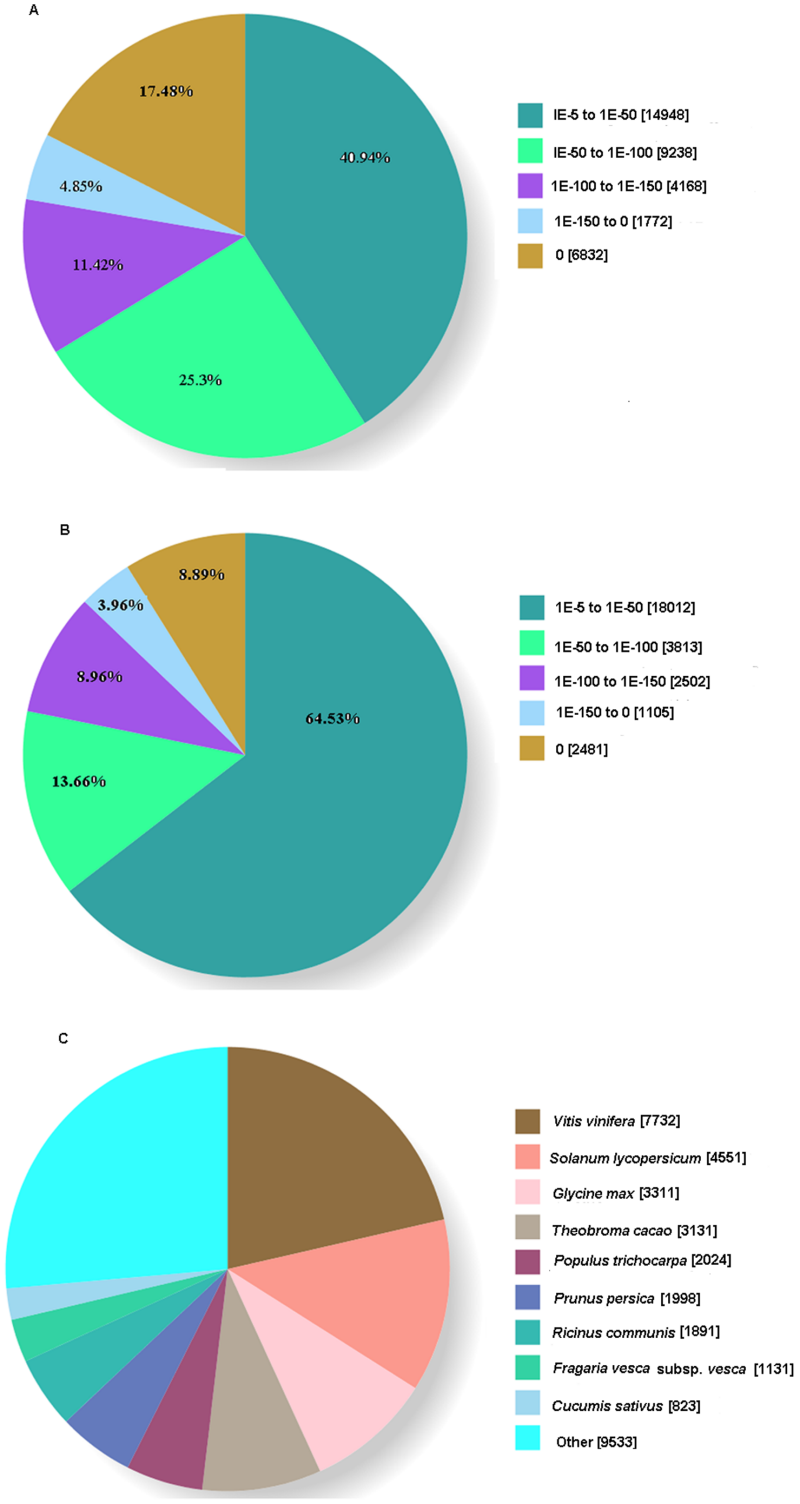
Characteristics of homology search of *Youngia japonica* unigenes. (A) E-value distribution of the top Blastx hits against the non-redundant (Nr) protein database for each unigene; (B)E-value distribution of the top Blastn hits against the NCBI non-redundant nucleotide sequence (Nt) database; (C) number of unigenes matching the top nine species using Blastx in the Nr database.

Among these aligned unigenes in Nr protein database, 59.06% unigenes had an E-value less than 1.0E^−50^ and showed very strong homology, whereas the remaining 40.94% unigenes had an E-value between 1.0E^−5^ and 1.0E^−50^ (Fig. 5A). The similarity distribution of aligned unigenes in the Nr database showed that 67% unigenes had a similarity higher than 60%, 26.48% unigenes had a similarity between 40% and 60%, and only 6.52% unigenes had a similarity lower than 40% (Table 4). Among these aligned unigenes in the Nt database, 27913 unigenes had an E-value less than 1.0E^−5^, and 64.53% unigenes had an E-value between 1.0 E^−5^ and1.0 E^−50^ (Fig. 5B). The similarity distribution of aligned unigenes in the Nt database showed that 86.69% unigenes had a similarity higher than 80%, only 6.76% unigenes had a similarity between 60% and 80%, and no unigenes had a similarity lower than 60% (Table 4). For the species distribution of aligned unigenes in the Nr database, approximately 73.61% were matched with sequences from nine dicotyledonous dicotyledonousdicotyledonousspecies, namely, *Vitis vinifera* (21.4%), *Solanum lycopersicum* (12.6%), *Glycine max* (9.17%), *Theobroma cacao* (8.67%), *Populus trichocarpa* (5.6%), *Prunus persica* (5.53%), *Ricinus communis* (5.23%), *Fragaria vesca* subsp. *vesca* (3.13%), and *Cucumis sativus* (2.28%) (Fig. 5C). In summary, homologous sequence annotation information of the majority of the unigenes could be obtained. The results also show that the accuracy of assembled transcripts was high, and our strategy was efficient.

**Table 4:** Similarity distribution of unigenes of *Youngia japonica* aligned in the Nt and Nr databases.

Similarity range	Number of unigenes (Nr)	Percent of unigenes (Nr)	Number of unigenes (Nt)	Percent of unigenes (Nt)
23%–40%	2382	6.52%	0	0.00%
40%–60%	9666	26.48%	0	0.00%
60%–80%	14144	38.74%	1887	6.76%
80%–100%	8240	22.57%	24199	86.69%
100%	2076	5.69%	1827	6.55%

The transcriptome of *Y. japonica* data consists of 51,850 unigene sequences, which is larger than that of either *Centaurea solstitialis* (40,407) or *Zinnia violacea* (20,767), but smaller than the EST collection of *L. sativa* (81,330), *Chrysanthemum nankingense* (70,895), and *C. arvense* (63690) (http://www.ncbi.nlm.nih.gov/dbEST/dbEST_summary.html) [6]–[10].

In biological processes, 18 unigenes were related to C4 photosynthesis (GO: 0009760) and 21 unigenes were related to phosphoenolpyruvate carboxylase activity (GO: 0008964). However, no genes related to ribulose-1,5-bisphosphate carboxylase/oxygenase were found. These results suggest that *Y. japonica* is a C4 plant. Three photosynthetic pathways are used by plants, namely, C3 (most plants use), C4, and Crassulacean acid metabolism (CAM). C3 is the ancestral pathway, whereas C4 and CAM were recently discovered. C4 plants evolved from C3 plants [31]. Thus, *Y. japonica* is one of the most evolved plants. The C4 pathway is attributed to the products of plants in warm-, light-, and moisture-limited conditions [32], [33]. C4 photosynthesis maybe used to resist adversity, and allows *Y. japonica* to evolve as a widely distributed weed species. The number of unigenes (COG classification) related to “carbohydrate transport and metabolism” and related to “energy production and conversion” were higher in *Y. japonica* (8.47% and 6.62%, respectively) than in Ma Bamboo (*Dendrocalamus latiflorus*) (8.19% and 5.3%, respectively) [34]. The fact that more genes were involved in “carbohydrate transport and metabolism” and “energy production and conversion” in *Y. japonica* than in Ma bamboo (C4 plant), suggested that *Y. japonica* possibly evolved toward a high utilization rate of energy. But further studies are required to prove this hypothesis.

According to GO classification, the unigenes were related to metabolic processes (76.65%), response to stimulus (56.86%), and biological regulation (52.22%). A total of 5,596 unigenes were related to biological processes, including defense response to fungus, virus, bacterium, etc. COG classification indicated that the number of unigenes related to “signal transduction mechanisms” was a little higher in *Y. japonica* (10.95%) than in Ma Bamboo (10.8%) (Liu et al. 2012), but was much higher than in *Chrysanthemum nankingense* (6.9%) [7] (Fig. 3). There were a high number of unigenes related to “response to stimulus” and “signal transduction mechanisms” in *Y. japonica*, which indicated that *Y. japonica* might response to environment stress very rapidly. Numerous unigenes (236) were related to defense response, which can also enhance the ability to resist adversity. There were 203 unigenes related to plant-pathogen interaction pathway (ko04626), and 39 unigenes of them with RPKM>10. Unigenes related to plant-pathogen interaction pathway possibly were also involved with numerous important physiological functions. For example, T1Unigene BMK.43981 was expressed with the highest level (RPKM = 500.34). The functions of this gene include biological process: very long-chain fatty acid metabolic process (GO: 0000038) and GTP catabolic process (GO: 0006184) and translational elongation (GO:0006414) and cuticle development (GO: 0042335); molecular function: translation elongation factor activity (GO: 0003746), GTPase activity (GO: 0003924), protein binding (GO: 0005515) and GTP binding (GO: 0005525); cellular component: nucleolus (GO: 0005730), cytosol (GO: 0005829), nucleoid (GO: 0009295), chloroplast thylakoid membrane (GO: 0009535), chloroplast stroma (GO: 0009570), chloroplast envelope (GO: 0009941) and apoplast (GO: 0048046). Secondly was T1 Unigene BMK.44184 (RPKM = 197.18). (Table S1), which was an allergen in *Olea europaea*, according to the Swissprot annotation. The functions of this gene includes molecular function: calcium ion binding (GO: 0005509) and metal ion binding (GO: 0046872); cellular component: nucleus (GO: 0005634), mitochondrion (GO: 0005739), vacuole (GO: 0005773), peroxisome (GO: 0005777) and plasma membrane (GO: 0005886); biological process: response to cold (GO: 0009409), double fertilization forming a zygote and endosperm (GO: 0009567), plant-type cell wall modification (GO: 0009827), pollen tube growth (GO: 0009860), regulation of flower development (GO: 0009909), root hair elongation (GO: 0048767) and regulation of nitric oxide metabolic process (GO: 0080164). Genes related to phenylpropanoid pathway (ko00940), terpenoid biosynthesis (ko00900), ubiquinone and other terpenoid-quinone biosynthesis (ko00130) (Fig. 4), which were induced by necrotrophic fungal infection in *Lactuca sativa*, were also found in *Y. japonica* [6]. These pathways were also possibly related to fungal resistance in *Y. japonica*. Furthermore, terpenoids are related to the antioxidant and anti-tumor activities of *Y. japonica* [3]–[5]. Interestingly, two unigenes were found related to bacterial invasion of epithelial cells pathway (ko05100). Despite the small number of genes, it is one of the evidences of secretory cells in response to bacterial infection. Obviously, genes related to anti-bacterial infection, defense and metabolic pathways exhibited diversity in *Y. japonica*.

Although only 595 unigenes (4.85%) related to secondary metabolites biosynthesis, transport and catabolism were found in COG classification in *Y. japonica*. Twelve pathways were found in KEGG classification (7.40% of the total 162 pathways). Eighteen unigenes were involved in diterpenoid biosynthesis (ko00904). T1 Unigene BMK.34073 was the most abundant ones (RPKM = 10.22). This gene involved with biological process: ent-kaurene oxidation to kaurenoic acid (GO: 0010241), which was related to ent-kaurene oxidase both in *Arabidopsis thaliana* and *Lactuca sativa*. This gene also has many other biological functions, including iron ion binding (GO: 0005506), electron carrier activity (GO: 0009055), heme binding (GO: 0020037), ent-kaurene oxidase activity (GO: 0052615), ent-kaur-16-en-19-ol oxidase activity (GO: 0052616), and ent-kaur-16-en-19-al oxidase activity (GO: 0052617). Forty five unigenes were related to flavonoid biosynthesis (ko00941), with 8 overrepresented (RPKM >25, Table S2). T1 Unigene BMK.14761 was the most abundant ones (RPKM = 391.82). This gene was found related to trans-cinnamate 4-monooxygenase in *Helianthus tuberosus* and *Cynara scolymus*. While in *Artemisia annua* (Sweet wormwood), it was related to Cytochrome P450 mono-oxygenase. This genes involved with many functions, including molecular function: iron ion binding (GO: 0005506), electron carrier activity (GO: 0009055), trans-cinnamate 4-monooxygenase activity (GO: 0016710), heme binding (GO: 0020037); cellular component: endoplasmic reticulum (GO: 0005783); plasma membrane (GO: 0005886), plant-type cell wall (GO: 0009505); biological process: pollen development (GO: 0009555), response to light stimulus (GO: 0009416), response to wounding (GO: 0009611), lignin metabolic process (GO: 0009808), growth (GO: 0040007), oxidation-reduction process (GO: 0055114) and response to karrikin (GO: 0080167). Nine unigenes were involved in flavone and flavonol biosynthesis pathway (ko00944), with four high level expressed unigenes (RPKM >10, Table S2). Twenty six unigenes were involved in isoquinoline alkaloid biosynthesis (ko00950), with 8 overrepresented unigenes (RPKM>10, Table S2). Thirty unigenes were involved in tropane, piperidine and pyridine alkaloid biosynthesis pathway (ko00960), with 8 overrepresented unigenes. In steroid biosynthesis pathway (ko00100, 34 unigenes), there were 11 unigenes with high express level (RPKM >10). Fifty two unigenes were involved in ubiquinone and other terpenoid-quinone biosynthesis (ko00130), with 7 overrepresented. In terpenoid backbone biosynthesis pathway (ko00900, 100 unigenes), 22 unigenes was overrepresented. Twenty six unigenes were involved in limonene and pinene degradation (ko00903), with 7 high expressed unigenes (RPKM >10, Table S2). Eight unigenes were related to brassinosteroid biosynthesis (ko00905). T1 Unigene BMK.9768 was with the highest expression level (RPKM = 85.35). This gene was involved with many functions, including biological process: polysaccharide biosynthetic process (GO: 0000271), multidimensional cell growth (GO: 0009825), positive regulation of flower development (GO: 0009911), cell tip growth (GO: 0009932), response to UV-B (GO: 0010224), brassinosteroid homeostasis (GO: 0010268), pollen exine formation (GO: 0010584), brassinosteroid biosynthetic process (GO: 0016132), anthocyanin accumulation in tissues in response to UV light (GO: 0043481), tapetal cell differentiation (GO: 0048657), root hair elongation (GO: 0048767), oxidation-reduction process (GO: 0055114), cell wall organization (GO: 0071555); molecular function: monooxygenase activity (GO: 0004497), iron ion binding (GO: 0005506), oxidoreductase activity, acting on paired donors, with incorporation or reduction of molecular oxygen (GO: 0016705), oxygen binding (GO: 0019825), heme binding (GO: 0020037), electron carrier activity (GO: 0009055); and cellular component: mitochondrion (GO: 0005739). This gene was related to Cytochrome P450 in *Arabidopsis thaliana*, while in *Artemisia annua*, it was putatively related to steroid 23-alpha-hydroxylase cytochrome P450. In stilbenoid, diarylheptanoid and gingerol biosynthesis pathway (ko00945, 33 unigenes), 14 unigenes were overrepresented ((RPKM >10). In phenylpropanoid pathway (141 unigenes), 22 unigenes were overrepresented. T1 Unigene BMK.43909 had the highest express level (RPKM = 950.97, Table S2). This gene was involved in many functions, including molecular function: peroxidase activity (GO: 0004601), heme binding (GO: 0020037), metal ion binding (GO: 0046872); biological process: response to oxidative stress (GO: 0006979) and oxidation-reduction process (GO: 0055114). This gene was related to peroxidase in *Cichorium intybus*, *Arabidopsis thaliana* and *Bruguiera gymnorhiza*.

Diversity of pathways and unigenes of secondary metabolites in *Y. japonica* suggest that secondary metabolites may play important physiological functions in *Y. japonica*. Some of the secondary metabolites are important plant hormones, such as gibberellins and brassinosteroids, which have an extremely important role on the growth of plants, development, aging and stress resistance. Studies of functional genes related to secondary metabolites will be one of the focus in adaptive evolution research of this species.

In the pathway of plant hormone signal transduction (ko04075, 297 unigenes), 77 unigenes were overprinted (RPKM >10, Table S3). T1 Unigene BMK.37824 was the most highly expressed gene. This gene was involved in many molecular function and biological process, including response to auxin stimulus (GO: 0009733) and response to abscisic acid stimulus, response to heat (GO: 0009408) and positive regulation of seed germination (GO: 0010030) and response to wounding (GO: 0009611), and release of seed from dormancy (GO: 0048838), etc. The other unigenes were also involved in regulating gene expression in development, cell differentiation, physiological and defense processes. For example, T1 Unigene BMK. 44869 was involved in many regulation of defense response (GO: 0031347) and T1Unigene BMK.44232 related to regulation of cellular process (GO: 0050794). Additional studies deploying accurate molecular and proteomic analysis procedures are required to validate these genes functions.

The 3,648 putative microsatellites identified in this study can be used for screening for population level diversity of molecular markers in future. The unigenes related to the low copy nuclear genes, may be used in designing primers and developing nuclear gene markers, which is important for the study of plant phylogeny. In addition, 476 unigenes (3.88%) were functional unknown. New functions could be identified for these genes, which are worthy of attention in the future study.

## Conclusion

It is the first time to study the most comprehensive transcriptome for *Y. japonica* from mixed tissues (flowers, leaves and stems) using the Illumina platform. This transcriptome analysis provided 51,850 unigenes, among which 70.42% were aligned to the Nr database. However, a reference genome sequence for *Y. japonica* was unavailable. A large number of candidate genes potentially involved in growth, development, flowering, plant hormone and defense responses are worthy of further investigation. C_4_ photosynthesis genes were involved the biological processes of *Y. japonica*. There were 595 unigenes (4.85%) related to secondary metabolites biosynthesis, transport and catabolism (12 pathways). These data provide insights into the genetic diversity of *Y. japonica*. A total of 3,648 SSRs were identified, which will benefit marker development. These data constitute a new valuable resource for genomic studies on *Youngia* and, more generally, Cichoraceae.

## Supporting Information

Table S1 **Unigenes related to plant-pathogen interaction pathway.**
(XLS)

Table S2 **Unigenes related to secondary metabolites biosynthesis, transport and catabolism pathway.**
(XLS)

Table S3 **Unigenes related to plant hormone signal transduction.**
(XLS)
